# Can they stomach it? Parent and practitioner acceptability of a trial comparing gastric residual volume measurement versus no gastric residual volume in UK NNU and PICUs: a feasibility study

**DOI:** 10.1186/s40814-021-00784-5

**Published:** 2021-02-16

**Authors:** Elizabeth Deja, Louise Roper, Lyvonne N. Tume, Jon Dorling, Chris Gale, Barbara Arch, Lynne Latten, Nazima Pathan, Helen Eccleson, Helen Hickey, Jenny Preston, Anne Beissel, Izabela Andrzejewska, Frédéric V. Valla, Kerry Woolfall

**Affiliations:** 1 Institute of Population Health, University of Liverpool, Liverpool, UK; 2 School of Health & Society, University of Salford, Manchester, M6 6PU UK; 3 Division of Pediatrics and Neonatal-Perinatal Medicine, Dalhousie University, Halifax, Canada; 4 Neonatal Medicine, School of Public Health, Faculty of Medicine, Imperial College London, Chelsea and Westminster Hospital campus, London, UK; 5 Medicines for Children Clinical Trials Unit, Clinical Trials Research Centre, University of Liverpool Institute of Child Health Alder Hey Children’s NHS Foundation Trust Liverpool, Liverpool, UK; 6 Department of Dietetics, Alder Hey Children’s Hospital, Liverpool, UK; 7 Paediatric Intensive Care, University of Cambridge, Addenbrooke’s Hospital Cambridge, Campbridge, UK; 8 Institute of Translational Medicine, University of Liverpool, Liverpool, UK; 9 Neonatal Intensive Care Unit, Hôpital Femme Mère Enfant, Hospices Civils de Lyon, Lyon-Bron, France; 10 Neonatal Intensive Care Unit, Chelsea and Westminster Hospital, London, UK; 11 Faculty of Health & Applied Sciences, University of the West of England, Bristol, UK; 12 Pediatric Intensive Care Unit, CarMEN INSERM UMR 1060 Equipe INFOLIP, Hôpital Femme Mère Enfant, Hospices Civils de Lyon, Lyon-Bron, France

**Keywords:** Gastric residual volume, Intensive care, Acceptability, Feasibility

## Abstract

**Background:**

Routine measurement of gastric residual volume (GRV) to guide feeding in neonatal and paediatric intensive care is widespread. However, this practice is not evidence based and may cause harm. As part of a feasibility study, we explored parent and practitioner views on the acceptability of a trial comparing GRV measurement or no GRV measurement.

**Methods:**

A mixed-methods study involving interviews and focus groups with practitioners and interviews with parents with experience of tube feeding in neonatal and/or paediatric intensive care. A voting system recorded closed question responses during practitioner data collection, enabling the collection of quantitative and qualitative data. Data were analysed using thematic analysis and descriptive statistics.

**Results:**

We interviewed 31 parents and nine practitioners and ran five practitioner focus groups (*n*=42). Participants described how the research question was logical, and the intervention would not be invasive and potential benefits of not withholding the child’s feeds. However, both groups held concerns about the potential risk of not measuring GRV, including delayed diagnosis of infection and gut problems, increased risk of vomiting into lungs and causing discomfort or pain. Parent’s views on GRV measurement and consent decision making were influenced by their views on the importance of feeding in the ICU, their child’s prognosis and associated comorbidities or complications.

**Conclusions:**

The majority of parents and practitioners viewed the proposed trial as acceptable. Potential concerns and preferences were identified that will need careful consideration to inform the development of the proposed trial protocol and staff training.

**Supplementary Information:**

The online version contains supplementary material available at 10.1186/s40814-021-00784-5.

## Introduction

Critically ill neonates and children who receive sufficient nutrition have reduced complications, spend less time on mechanical ventilation, reduced time in intensive care and have overall improved health outcomes [1–5]. Nutrition should, if at all possible, be delivered enterally to maintain gut barrier function [6]. On average, children in paediatric intensive care units (PICUs) receive less than half of their nutritional requirements [2, 7]. In preterm infants, feeds are gradually increased to the immature, naïve gut and clinicians closely observe feed tolerance and for signs of complications such as necrotising enterocolitis [8].

One of the practices that contributes to children not receiving adequate enteral nutrition is the routine measurement of the gastric residual volume (GRV) (fluid in the stomach) to guide both the initiation and progression of enteral feeds [9]. Commonly cited rationales for measuring GRV are to assess ‘feed tolerance’, to prevent complications such as vomiting from a full stomach with subsequent pulmonary aspiration and in neonates, to detect necrotizing enterocolitis (NEC) [10]. Large GRVs almost always result in withholding or reducing of feed volumes [11–13]. However, what is considered a ‘large’ GRV measurement and clinical response to this measurement is extremely variable [14]. Importantly, this measurement is not considered accurate [15–17] and has not been shown to reflect delayed gastric emptying [18].

Clinical trials in the neonatal and paediatric critical care setting are challenging to conduct due to ethical and practical considerations [19–22]. In line with the Medical Research Council’s framework for complex intervention development [23] conducting trial feasibility studies can help mitigate these challenges and establish whether the trial is acceptable to parents and practitioners, and practical to conduct [24, 25]. As part of a pre-trial feasibility study [14, 26], we explored parent and practitioner perspectives on the acceptability of conducting a clinical trial comparing no routine GRV measurement (the intervention) to routine GRV measurement in UK neonatal intensive care units (NNU) and paediatric intensive care unit (PICU) settings.

## Methods

### Study design

A mixed-methods design involving interviews and focus groups, incorporating a voting system to enable the collection of both qualitative and quantitative data. Our previous research, relevant literature and pre-study patient and public involvement guided the development of the topic guides and participant information [27–29]. We explored parent and practitioner views on the acceptability of the proposed GASTRIC trial including acceptability of measuring or not measuring GRV, willingness to participate in a future trial, trial information materials, barriers and facilitators to trial recruitment (S1, example parent topic guide).

### Participant recruitment

#### Parent recruitment

Based on our previous studies [27, 29–31], we anticipated that 20–30 parents (*n*=10–15 in each setting) would be needed to reach data and thematic saturation, the point where additional data does not lead to any new major themes during analysis and the researcher notes high levels of ‘information redundancy’ during data collection [32–34]. Parents of children with experience of tube feeding in NNU and/or PICU in the last 3 years were recruited through four routes: (1) social media or website advertising [including Twitter (URL: www.twitter.com; Twitter, Inc., San Francisco, CA, USA), Bliss (URL: www.bliss.org.uk), Sepsis Trust (URL: https://sepsistrust.org), hospital charities and Mumsnet (URL: www.mumsnet.com)], (2) targeted emails to study team contacts and a database from our previous research [29], (3) an advert in a national newspaper and (4) word of mouth (e.g. research team primary school networks).

#### Practitioner recruitment

We aimed to hold four focus groups (two NNU, two PICU) in different geographical locations across the UK. We also purposively targeted individuals and groups to take part in a telephone interview to insure all key professional groups were included (e.g. doctors, nurses, dietitians and surgeons). We recruited through e-mail invitations and postings on networks known to the co-applicants of the study including the Paediatric Intensive Care Society and British Dietetics Association.

### Conduct of interviews and focus groups

#### Parent interviews

Psychologists LR and ED (PhD, female research associates) responded to parents’ requests to participate in sequential order, confirmed eligibility and emailed them the draft trial participant information sheet (PIS) (S2, PIS). A telephone or face-to-face interview was arranged depending on geographical location and participant preference. There was no relationship between the researcher and participant prior to the interviews. After interviews participants were sent a £30 shopping voucher to thank them for their time.

#### Practitioner focus groups and interviews

At the start of the focus group or interview, the researcher (ED, LR) checked that participants had read the PIS. Eleven closed questions were administered in focus groups using a voting system (Turning Technologies, Youngstown, OH, USA) and administered verbally during interviews. This allowed for the collection of staff demographic information, to insure data collection from all staff on key questions and facilitate discussion. As part of an iterative approach, we added or amended questions in the parent and practitioner topic guides as interviewing and analysis progressed (see S3, practitioner topic guide) [35]. Audio-recorded verbal or written consent was sought before interviews or focus groups began. Interviews stopped when saturation point was reached. All digital audio recordings were transcribed verbatim by a professional transcription company (Voicescript Ltd., Bristol, UK). Transcripts were anonymised and checked for accuracy.

### Data analysis

LR and ED led the qualitative analysis with assistance from KW (PhD, sociologist). Analysis used a thematic analysis approach [36–38] to examine patterns within the data related to views on trial design and acceptability (see S4, Table 1). Analysis was broadly interpretive, iterative and used a combination of inductive and deductive approaches to coding [39, 40]. NVivo 10 software (QSR International Pty Ltd., Melbourne, Australia) was used to assist in the organisation and coding of data. Quantitative data from closed questions were entered into SPSS Version 20.0 (IBM Corp., Armonk, NY, US). Descriptive statistics are presented with frequencies and percentages. Synthesis of qualitative and quantitative data drew on the constant comparative method [41, 42]. This involved ED and LR looking across quantitative and qualitative themes and quantitative output for themes/data output related to trial acceptability***.*** Analysis was interpretive- theorising the significance of the patterns and their broader meanings and implications. A final stage of analysis involved consideration of both qualitative and quantitative findings against the Theoretical Framework of Acceptability (TFA) [25] to help conceptualise and discuss the overall acceptability of the proposed trial. The TFA is designed to assist researchers in assessing the acceptability of healthcare interventions, including clinical trials. The TFA presents seven theoretical constructs for researchers to consider when assessing whether people delivering or receiving a healthcare intervention consider it to be appropriate.

## Results

### Participants—parents

**Fig. 1:**
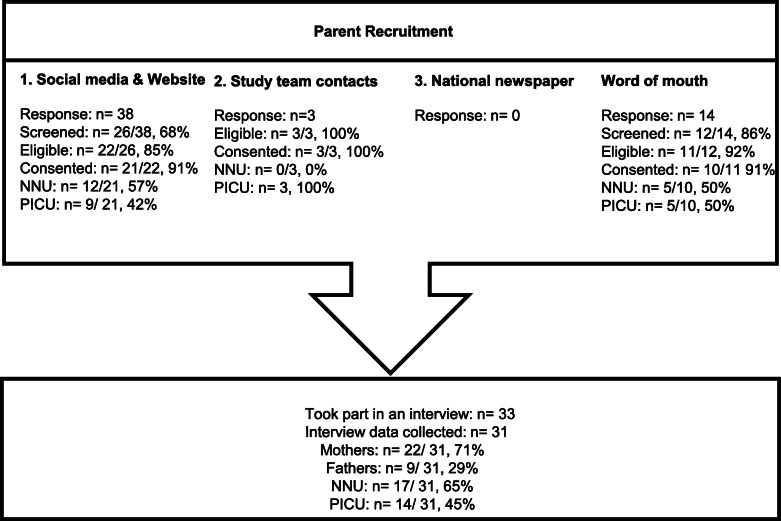
Parent recruitment

Fifty-four parents registered interest, of whom 38 were interviewed (Fig. 1). Two PICU parents were interviewed but not included in the analysis due to recording equipment failure. Saturation was reached both within and across the NNU and PICU samples. Our final sample of 31 included 17 NNU and 14 PICU parents including 22 mothers (12 NNU, 10 PICU; 4 were bereaved) and nine fathers (5 NNU, 4 PICU; 1 bereaved). Interviews took place between May and November 2018. Seventeen NNU parent interviews related to 19 children (Table 1) and 21 different hospitals. Three mothers had twins who were both admitted to NNU, and one set of parents were interviewed separately regarding the same child. Fourteen PICU parent interviews related to ten children, with four sets of parents interviewed separately regarding the same child. Six of the ten children had also been admitted to NNU at birth and five had had multiple PICU admissions. Interviews took place on average (mode) 11 months (range=0.8–37 months) from hospital admission and took on average 68 (SD=12.7) minutes. Interviews took place between May and November 2018.

**Table 1:** Child characteristics and NNU/PICU admission information based on parent interview accounts

Characteristics	NNU	PICU
Child age at hospital admission (or birth)	Median 29 weeks of gestation (range: 24–41 weeks)	Median 8-month old (range 3 weeks–12 years)
Days in unit	Median 21 (range: 1–140, missing *n*=1)	Median 8 (range 2–72)
Days in hospital	Median 57 (range: 7–152)	Median 39 (range 3–196)
Days on feeding tube	Median 58 (range: 2–210)	Median 127.5 (range: 5–547)
Days on breathing support	Median 56 (range: 0–370)	Median 6 days (range 0–168)
Main reason for admittance	Prematurity (*n*=18) Meconium aspiration syndrome (*n*=1)	Heart conditions, e.g. congenital heart defect, hyperplastic left heart (*n*=4) Sepsis (*n*=2) Reconstruction of airway (*n*=1) Complications linked to chronic conditions, e.g. holoprosencephaly, Noonan’s syndrome, prematurity (*n*=3)

### Participants—practitioners

Fifty-one practitioners took part in either a focus group or interview. Five focus groups were conducted at three UK hospitals, and this included two in NNU and three in PICU. Forty-two practitioners (16 NNU, 26 PICU) participated in one of the five focus groups. Sixty-two percent (26/42, 62%) of focus group participants were clinical nurses. The remainder were research nurses (5/42, 12%), senior doctors (3/42, 7%) and dietitians (2/42, 5%). Six participants (6/42, 14%) categorised themselves as ‘other’. Three were student nurses, three did not specify.

As the majority of focus group participants were nursing staff, we purposively contacted ten consultant level doctors’ doctors, three surgeons and five dietitians and invited them to take part in a telephone interview. Five additional dietitians expressed interest in being interviewed after receiving study information through their professional networks. Of those we directly targeted, 12/21 (58%) did not respond. A total of nine interviews were conducted including three consultant doctors (2 NNU, 1 PICU), four dietitians (1 NNU, 3 PICU) and two surgeons (both surgeons worked with NNU and PICU patients).

Ten UK hospitals were represented in the combined interview and focus group sample. The majority had experience of conducting paediatric clinical trials (39/51, 76%). Focus groups took on average 55 min (range 49–68 min) and telephone interviews 32 min (range 26–45 min).

### Parents’ views on GRV measurement and feeding

Interviews began with a discussion of parents’ NNU/PICU experience, including how they prioritised feeding as a component of critical care and their knowledge of GRV. Twenty-one parents (68%) recalled their child’s GRV being measured (15 NNU, 6 PICU). Those who did recall GRV being measured had varying understanding of its utility:

I remember quite a few occasions where they were, erm, sort of like take it out, work it out if there was too much left then they wouldn’t do the next one as much. (P21, Mother, PICU)

Parent’s views around GRV measurement appeared to be linked to their views on the importance of feeding in the ICU, their child’s prognosis and associated comorbidities or complications. For example, parents of children who experienced an imminently life-threating condition, such as sepsis, had not considered feeding to be a priority in the ICU:

Well initially obviously they were trying to keep her alive (P24, mother, PICU).

In contrast, parents of premature children or those with chronic conditions, such as a congenital heart defect, viewed feeding as very important. Weight gain and calorie intake were seen to have direct causal relationship with short and longer-term outcomes:

We knew that gaining weight was central to them going home and we also knew that the greater their weight, the less risk there was of an infection. (P05, father, NNU)

Parents described how they were initially unaware of how difficult, yet important, establishing feeds was at the beginning of their NNU stay and would still be in what feels like an acute situation:

I remember them saying, oh, they’ve coped really well with the last feed, and you think, coped really well? How hard is eating? And they don’t explain to you that it can have a huge a huge impact (P11, mother, NNU).

Over half of the PICU parents had past NNU experience and therefore had already developed views on feeding in the early phase of their PICU stay, which would be the point at which parents would be approached to discuss their child’s participation in the proposed trial.

### Potential benefits and concerns about conducting the proposed GASTRIC randomised control trial

Parents and practitioners were asked to consider the proposed trial. Practitioners identified some potential benefits of not measuring GRV such as reduced reliance on intravenous lines and less problems with gut motility. Both parents and practitioners described how not measuring GRV might be beneficial because it might increase nutritional intake and remove a potentially unnecessary and “quite invasive” (P03, mother, NNU) intervention that may increase the “risk of infection” (P03, FG3, nurse, PICU). However, as Table 2 shows, 88% of practitioners felt that there would be barriers to staff not measuring GRV in the proposed trial.

**Table 2:** Practitioner views on ‘Do you think there will be any barriers to staff not measuring GRV?’ in a proposed trial by role (*N*=48)

	Senior nurse (***n***=11)	Junior nurse (***n***=15)	Research nurse (***n***=4)	Senior doctor (***n***=7)	Dietitian (***n***=7)	Other (***n***=4)	Total
Yes	9 (21%)	14 (33%)	4 (10%)	5 (12%)	6 (14%)	4 (10%)	42 (88)
No	2 (33%)	1 (17%)	0 (0%)	2 (33%)	1 (17%)	0 (0%)	6 (12)

Practitioners and parents voiced concerns about how not measuring GRV may delay the diagnosis of bowel or stomach problems, infection, NEC, or lead to incorrect feeding tube placement or feed intolerance:

You’ll have a baby who you’re not measuring residuals anymore, who will end up with NEC and then they’ll say oh this would have been picked up earlier if we'd realised it has bilious aspects. (P05, FG5, consultant neonatologist, NNU)

Nevertheless, half of the practitioner sample (mainly doctors) interviewed also described how they:

Don’t think that measuring the GRV is going to be a, um, reliable indicator of whether a baby’s got NEC or is at risk of getting NEC (P05, interview, surgeon).

With others stating that measuring GRV was, on its own: “completely meaningless” (P06, interview, doctor, NNU) and that:

Babies who are gonna develop an important pathology, never present purely with gastric residuals (P06, interview, doctor, NNU).

Other concerns, such as not detecting incorrect tube placement, or not being able to wind a child, were addressed by researcher clarification that children would still have routine gastric aspirates assessed for tube position (but not assessed for gastric residual volumes) in line with existing guidance.

Parents and practitioners were also worried about the risk of vomiting into the lungs leading to possible chest infections and breathing difficulties. Interestingly, some suggested contrasting views in that both measuring and not measuring GRV may lead to vomiting or cause pain and discomfort. Whilst some parents and practitioners were concerned that returning stomach contents may cause a child to vomit: “nine times out of ten if they shot it back in she was sick.” (P15, NNU, father) or cause pain or discomfort. Others felt that not measuring GRV may result in discomfort and vomiting.

### The challenge of changing routine clinical practice

Almost half the practitioners, unprompted, 23/51 (45%) discussed the potential difficulty of changing such an accepted and embedded routine practice for a clinical trial:

I can imagine there will be barriers to it because it’s just the way that things are done (P02, interview, dietitian, PICU).

Nurses, in particular, valued GRV measurement as a useful common practice that they perceived informed patient care. Six participants, from a range of clinical backgrounds, described how they would “feel uncomfortable” (P06, FG3, senior nurse, PICU) about changing this ‘normal’ practice for a trial: “without the evidence” (P07, FG2, research nurse, PICU) to support such a change. Others stated that while they would personally participate in the proposed trial, they felt that their colleagues may not be willing to change their behaviour:

If the surgeons are involved, they'll want to know what aspirates are and the amount as well (P05, FG1, nurse, NICU).

Some dietitians stated that they were planning to change their practice to not measure GRV, which meant the proposed trial was less of a concern to them. Suggestions included the need for bespoke site training to assist engagement and behaviour change, including additional information to support the study rationale, including evidence to:

Demonstrate why not measuring GRVs would be a sensible thing to do and in fact might be beneficial (P05, interview, surgeon).

### Overall views on the acceptability of the proposed GASTRIC randomised control trial

Parents and practitioners were asked to reflect and consider the acceptability of the proposed trial. Despite the concerns identified, the majority (39/46, 85%, 5 missing) of practitioners indicated it would be acceptable ‘to conduct the proposed trial’. Only 15% (7/46) said it was not acceptable to conduct. Of these seven, six were junior nurses. Parents also supported the proposed trial, with 90% (28/31) stating that they would provide consent for their child to take part. This acceptability was underpinned by a belief that the proposed study question: ‘makes perfect sense’ (P23, father, PICU). Overall, both groups viewed the proposed ‘measuring GRV arm’ acceptable because it is a useful standard practice. For the parents, hospitals would: ‘Just carrying on doing what we’re doing anyway’ (P20, mother, PICU). There was some ambivalence amongst clinical staff about the importance of the proposed trial. Four doctors and one surgeon reflected that GRV measurement was not a: ‘*big issue*’ (P06, interview, doctor, NNU) or not important: ‘in the grand scheme of things’ (P07, interview, doctor, PICU) (Table 3).

Parents with experience of tube feeding at the point of their child’s intensive care admittance (*n*=11) appeared to have a trial arm preference. However, the trial arm (GRV or No GRV) they preferred varied. Although many stated that their preference would not prevent them from consenting to the trial, they would require detailed information about the potential risks and benefits of each trial arm to reach a consent decision. Conversely, parents with no pre-existing knowledge or beliefs about tube feeding viewed GRV measurement/no measurement as of little importance. This view point was potentially influenced by their view that feeding was a low priority during a critical care situation. These parents described how their child’s acute condition was the main priority, and a trial involving measuring or not measuring GRV would be therefore of low risk and therefore acceptable.

**Table 3:** Findings mapped against the Theoretical Framework of Acceptability

Group & data collection method	Affective attitude	Burden	Ethicality	Intervention Coherence	Opportunity Costs	Perceived Effectiveness	Self-Efficacy
**Parents** Interviews	28/31 (90%) would hypothetically provide consent for their child to take part if they were approached about the trial.	The intervention of not measuring GRV is less invasive than conducting GRV *“I just think that extra intervention, if it’s not actually doing anything positive, then is it really necessary*” (P18, mother, PICU & NNU) Conversely measuring GRV was also seen as acceptable as their child would be receiving normal or ‘standard’ clinical care as most units in the UK measure GRV: “Well that's just the way it is.” (P20, mother, PICU)	Belief it is important to help other children in the future. Important not to put your own child in additional risk or discomfort.	The proposed study question “*makes perfect sense*” (P23, father, PICU). The draft participant information sheet is “*very clear*” (P23, father, PICU), “*very straightforward*” (P01, mother, NNU) Suggested further improved by summarising key points and more detail on benefits.	NNU parents were concerned about the risk of delayed diagnosis of bowel or stomach problems, or missing signs of an infection. NNU and PICU parents worried about the risk of vomiting into lungs, “*nine times out of ten if they shot it back in, she was sick*” (P15, NNU, father) Both groups of parents focused on the risk of increased pain or discomfort.	When study rationale was explained parents understood how not measuring GRV might: -Reduce infections, -Improve overall health *“if they’re getting calories on board quicker, they’ll start to feel better quicker”* (P09, mother, NNU) -Reported reduced discomfort and pain.	The intervention was something parents understood and said they could support Conversations about research when their child was still critically ill: “would have to be very carefully approached” (P15, father, NNU). To prevent them from saying no due to situational incapacity.
**Practitioners** Focus groups and interviews	Of the forty-six practitioners (95.8%) who answered the question, ‘how acceptable is it to conduct the proposed trial?’ The majority, 84% (n=39/46) indicated it was ‘acceptable’ or ‘very acceptable’	One arm [measuring GRV] standard care. Less invasive	Not ethical to put a child at additional risk. It is not ethical to conduct unnecessary procedures. Consistent evidence based practice is important.	Generally a clear understanding of the proposed trail. However a few were confused about the difference between checking the gastric residual volume, by aspirating the entire stomach contents, and simply confirming the position of the feeding tube (by testing the pH) involving testing a small amount of fluid.	Concerns about increasing the risk of adverse events (AE), causing discomfort and pain or vomiting. not identifying early signs of: infections, gut obstructions or feed intolerances, lung injury (ARDS) NEC, stenosis of pyloric sphincter. In contrast, some practitioners stated that measuring GRV was, on its own, “completely meaningless” (P06, interview, doctor, NNU)	There were mixed views on the importance of the clinical question. However they highlighted the value of increasing “*nutritional intake by not checking [GRV], in the majority of patients”* (P02, interview, dietitian, PICU) and reduced risk of hospital related infection	All practitioners said that the trial was practically possible to conduct Six participants, from a range of clinical backgrounds, described how they would “*feel uncomfortable*” (P06, FG3, senior nurse, PICU) about changing this ‘normal’ practice for a trial: “*without the evidence*” (P07, FG2, research nurse, PICU) to support such a change.

I think that would be the last thing on me mind. So no, it wouldn't bother me. (P27, mother, PICU & NNU).

## Discussion

This study provides insight into the acceptability of the proposed trial of no routine GRV measurement by exploring views of parent’s and practitioners with relevant experience. Whilst studies have looked into parental views of tube feeding in the home environment [43–46], no previous research has explored parent or practitioner views on feeding or GRV in in neonatal or children’s intensive care. Overall, the majority of participants viewed a trial of GRV measurement compared to no GRV in NNU and/or PICU settings to be acceptable.

The Theoretical Model of Acceptability [25] provides a framework to help researchers unpack the multifaceted construct of acceptability, with seven components to consider. Our data suggest that the GASTRIC trial met five of the seven constructs [25]. The constructs of ‘opportunity costs’ and ‘self-efficacy’ were not fully met, which both relate to changing behaviours to engage with delivering the intervention. Parents and practitioners were concerned about the risk of delayed diagnosis of complications/problems by not measuring GRV, as well as increased vomiting or pain. A key challenge is the need for practitioners to change what is an accepted routine practice. Nurses valued GRV measurement as a useful practice that they perceived informed clinical decision making. This practice may provide nurses with a sense of security, in a practice-based profession where ‘doing things’ is highly valued [47]. Yet, the accuracy of this practice is heavily criticised for providing inaccurate values for a multitude of reasons. A previous study of nurses’ decision-making around this practice [10] showed most nurses frequently confused this practice with that of aspirating a small amount of fluid to confirm correct feeding tube placement through pH measurement. These concerns require careful consideration and a targeted education package to help facilitate practitioner engagement, particularly as some clinical staff appeared to be ambivalent about the importance of the proposed trial. Embedded research within the early stages of a GASTRIC trial [29, 48] will help establish whether this training is effective in assisting equipoise, influencing perspectives and practitioner behaviour change.

Parents’ views were often linked to their past experiences of feeding in the ICU, their child’s prognosis, associated comorbidities or complications and consequently duration of time on a feeding tube. This is particularly important, as views on feeding and GRV appeared to influence parental decision-making about trial acceptability. Therefore, in order to tailor trial discussions, practitioners should firstly explore whether parents have any previous ICU experience, the nature of the child’s illness and information that may be prioritised in the clinical setting.

The main limitation of this study is its hypothetical nature. The findings related to the importance placed on feeding, perceptions around measuring GRV and risk assessment around medical factors were substantially influenced by experiential knowledge. This is knowledge that the parents we interviewed had, but NNU parents at the point of being approached about the proposed GASTRIC trial may not. This is also true, but to a lesser existent, with the PICU parent sample who were more likely to have past tube feeding experience.

The strengths of this study lie in its large sample, with a range of views and experiences gathered from both parent and practitioners across the UK, and from different units with relevant experience. This insight enables the development of a study protocol that can aim to address potential challenges from the outset. In addition, there is evidence to show that drawing on findings from prospective acceptability studies’ to refine trial design and practitioner training can have a substantial impact on final acceptability [24, 29, 48].

## Supplementary Information

**Additional file 1: S1**. Example parent topic guide. **S2**. Draft Participant Information Sheet sent prior to interview. **S3**. Staff focus group topic guide. **S4 Table 1**. Approach to qualitative data analysis

## Data Availability

The datasets generated during and/or analysed during the current study are not publicly available as consent was not sought for data sharing.
