# Supplementary material for: Can they stomach it? Parent and practitioner acceptability of a trial comparing gastric residual volume measurement versus no gastric residual volume in UK NNU and PICUs: a feasibility study
Source: Pilot Feasibility Stud. 2021 Feb 16;7:49. doi: 10.1186/s40814-021-00784-5 (PMC7885383; doi:10.1186/s40814-021-00784-5)
Supplement: Supplementary file 1 — Additional file 1: S1. Example parent topic guide. S2. Draft Participant Information Sheet sent prior to interview. S3. Staff focus group topic guide. S4 Table 1. Approach to qualitative data analysis [file 40814_2021_784_MOESM1_ESM.docx]

**S1 GASTRIC example topic guide Questions**

| **Section 1-** **Knowledge and experience** | | |
| --- | --- | --- |
| 1.1 | My notes from when you registered interest in taking part in this study state that your child received hospital treatment in PICU in (*insert month and year*). Is that correct? (so that makes it X number of weeks ago)  How old was your child? *If premature ask for gestation in weeks at birth,* How is [*child name*] now?  What hospital was this? (prompt- explore if transferred)  Has he/she recovered from his/her PICU visit? | |
| 1.2 | Please could you give me an outline of what happened for them to need treatment in PICU?  How long was your child on a breathing support (differentiate between mechanical ventilation/CPAP/High flow)?  How long did they stay in intensive care unit part of the unit (differentiate not high dependency care e.g. HDU bed)?  How long were they in hospital? *Prompts: days in ICU, days in HDU, days on a ward) (terminology may vary).*  *Did they suffer from any serious bowel infections eg NEC?* | |
| 1.3 | Did they have a feeding tube in? , if yes when? Did they get tube fed whilst they were on the ICU?  Did the nurses ever talk to you about tube feeding your child?  *Prompt*: what did the nurse talk about to you about?  When was it first mentioned?  Was you childs feeding, for example getting enough food/calories, something that you thought about during their intensive care stay?  *Prompt:* How important was your child’s feeding to you during their  Intensive care stay?  Please describe any concerns you had during their time in PICU. *Prompt*: Were you concerned about whether your child was getting enough calories when they were being tube fed?  Do you remember the nurses finding out how much food was left in your child’s stomach (using a syringe to draw out any milk from their stomach)?  If you do, did you understand why they did this? Did they ever talk to you about this? If yes, What did they say?  Did your child ever have vomiting associated with tube feedings?  Were any specific clinical concerns about tube feeding and calorie intake, including whether tube feeding was stopped and why this happened e.g. weight not increasing, or procedures being delayed until feeding established etc | |
| 1.4 | **We are in the process of developing a type of study called a clinical trial. Have you ever heard of a clinical trial before?**  Explain: This is a type of medical research which provides information on the safety and effects of a drug or medical device (for example, a needle or line used to administer drugs). They are used to find out the best way to treat patients in the future. Trials are carried out to test many different medicines or treatments for children. Some test medicines or treatments which we think will be low risk, because doctors have already been using them for some time. Some trials test new medicines or ones where we don't know exactly what effects they will have in a particular situation.  **Have you ever been asked if your child could take part in a clinical trial?** If yes:  could you tell me a little more about that?  What was the trial looking at?  At what point where you approached and asked if you would give consent for your child to take part in the trial?  Could you tell me a bit more about what happened?  How did you feel at the time? | |
| **Section 2: Approach to consent in GASTRIC** | | |
| As described in the information sheet,  Children in intensive care often struggle to get even half of the amount of calories they need. Having enough calories is important when a child is sick to ensure they have enough energy to breathe and get better~~.~~ There are many reasons why children do not get enough calories, but one possible cause is the regular checking of how much fluid is in a child’s stomach by drawing out contents (e.g. milk) through a tube (called gastric residual volume, or GRV).  Nurses do this as they are concerned that too much fluid may build up in the child’s stomach and cause vomiting and potentially inhaling of this fluid into the lungs (causing pneumonia). However, these concerns have not been proven to be true. GRV measurement can lead~~s~~ to nurses stopping a child’s feeds. This GRV practice happens in all PICUs in the UK, but in some countries (eg. France) they stopped doing this many years ago. They probably stopped because three studies in adults in intensive care showed that if they did not measure this, the adults received more calories and there was no harm associated with doing it. So we believe that we should do a study in children (as they are not the same as adults) to see if this is the case and it might be better not to do this. We would appreciate your help in making sure this study is appropriate to families. | | |
| **Prompt: *Talk through the draft PIS emphasising:*** | | |
| 2.1 | | **- Read GASTRIC aims and objectives**  **- Discuss risks and benefits (section 2)**  **- Place in context of the trial:**  *Prompt:* Is each of these sections clear? Is their anything we need to add/ change?  Imagine you were a parent whose child had been admitted to the PICU because they are seriously ill (and the child required feeding using a tube). A nurse then approaches you when your child’s condition had stabilised. The nurse gives you this information sheet and goes through all of the information.  What would you think if someone approached you about a study like this, explained to you whilst your child was in PICU?  What would be your initial thoughts about this trial?  When do you think it would be best to be approached? (within 12/24/48 hours?) |
| 2.2 | | Would you have any concerns about the GASTRIC study? (*Prompt: after exploring concerns-*  Would you raise these concerns with the nurse or doctor? |
| 2.3 | | Would you have any questions about the GASTRIC study? |
| 2.4 | | Looking at the information sheet, are there any parts of the study design that you think parents may find difficult to understand? |
| 2.5 | | Are there sections of the information sheet which you would prioritise when making your decision about whether or not to consent?  *Prompt:*  were there any parts of the information sheet that stood out to you in terms of influencing your decision as to whether or not you would like to take part? |
| 2.6 | | How much time would you need to consider the information before making a decision about the GASTRIC Trial? |
| 2.7 | | Do you think you would consent (agree to allow your child to take part)?  Could you tell me a bit more about your reasons for this? |
| 2.8 | | What things do you think might stop you from agreeing to take part in a trial like this? |
| 2.9 | | What information would you like to know before you made a decision about whether to let your child participate? |
| 2.10 | | When do you think is the best time to approach a family to discuss the GASTRIC study? (*Prompt: we are suggesting within 24 hours)* |

**S2 Draft Participant Information Sheet sent prior to interview**

**Is it better to NOT measure gastric residual volume in tube-fed babies in intensive care?**

**The GASTRIC Study**

Insert NHS logo

<Trust address 1> <Trust address 2>

<Trust address 3> <postcode>

<telephone number>

Centre Number: <centre number>

**Parent/Carers Information Sheet**

**We invite you to take part in research study**

- In babies who are born very early, a long tube (called a drip or cannula) is placed into a babies large vein, and the baby is initially started on liquid food into the vein (called intravenous feed). As soon as possible, we start to give milk through a tube into the stomach, but it often takes a long time (days or weeks) for them to get enough of the feed to be able to stop the intravenous food and remove the drip. This is important because as soon as the baby reaches the full amount of feeds they need, through the stomach tube, can drip can come out. This drip with liquid food is always a risk of infection in babies, so the sooner we can get it out, the better. There are many reasons why babies take some time to reach their full amount of feeds by the tube feeding, but one routine practice that may be contributing to this, is the regular measuring of the baby’s stomach contents to see if they are coping with their tube feed. This is called the gastric residual volume or GRV. Measuring this often leads to nurses stopping the baby’s feeds based on a variable amount or the colour of the fluid coming from the stomach.
- This clinical trial (called GASTRIC) is designed to find out whether NOT routinely measuring the baby’s stomach contents (called GRV) during tube feeding is safe and beneficial.

| **Contents** | **Page** |
| --- | --- |
| 1) Why are we doing this study? | 2 |
| 2) Who can take part? | 2 |
| 3) How can I take part? | 2 |
| 4) Who is involved in  this study? | 3 |
| 5) What if there is a problem? | 3 |

**How to contact us**

**If you have any questions and/or would like to take part in an interview, please contact:**

<*Insert researcher name*>

Telephone: <*insert* *number*>

Email: <*insert study email address*>

**or**

Lead Investigator – Dr Kerry Woolfall

Telephone: 0151 794 4634

Email: [K.Woolfall@liverpool.ac.uk](mailto:K.Woolfall@liverpool.ac.uk)

Further information can be found on our website:

or Facebook page*:*

**1) Why are we doing this study?**

Babies in neonatal intensive care cannot feed normally by mouth and require feeding into their stomach via a tube (a naso-gastric tube). Although we try and start these feeds via the stomach as soon as we can, it often takes some time to reach the full amount of feeds the baby needs via the stomach, and during this time the baby will have a large tube (called a drip or cannula) in and be receiving liquid food via into a vein. Across the UK, a common practice is for nurses to pull back on this feeding tube regularly and gently suck out the baby’s stomach contents (the gastric residual volume or GRV) to try and see how well the baby is digesting their feed. However, this practice is variable across the UK and some countries (France) it is rarely measured in these babies. We therefore want to see whether it is safe and allows the baby to reach their full amount of feeds via the stomach quicker, if we do not measure GRV routinely. We expect to enrol around children in neonatal units across the UK in this study over 3 years

**2) Why has my baby been chosen?**

Your baby is in intensive care and requires feeding via a tube into their stomach (a naso-gastric tube).

**3) What will happen if I allow my baby to be in this study?**

If you decide to allow your baby in this study, they will be allocated at random to receive either usual care (regular measurement of GRV every hour or every few hours) or no routine GRV measurement. If they receive no GRV, they will be tube fed as per the usual neonatal unit guidelines but the stomach contents ( GRV) will not be measured and so feeds will not be stopped based on this. If your baby starts to vomit or shows other signs of feed not coping with their feeds, only then will the feed will be stopped.

**4) Are there any risks or benefits to my baby being in this study?**

Two small studies have not shown any additional risks with not measuring GRV in these babies and in some countries it is not routinely measured in many neonatal units (France). Your baby has to be given food via the stomach tube whilst in intensive care, but we do not know whether NOT measuring GRV is better and will lead to them reaching their full amount of feeds quicker. The risks considered to be possible with not measuring GRV tare that the baby would develop a severe bowel infection (called NEC) which may go unnoticed. Babies develop this sometimes anyway whether they are fed or not, and two studies have not proven that not measuring GRV affects this, but the babies in both studies did get to full feeds earlier. We cannot guarantee any benefit by your baby being in the trial, but there will be more detailed observation of your baby’s feed intake, along with other things such as the time they spend on the breathing machine, but we cannot guarantee this will benefit your baby.

**5) Do I have to allow my baby to take part and can I change my mind?**

It is entirely up to you whether you allow your baby to be in the study. If you do agree to your child being in the study, at any point, should you wish to withdraw them from the study; you are completely free to do so at any time, without giving a reason. This will not in any way affect the care that either you or your baby receives.

**6) What happens when this research stops?**

After your baby has been discharged from intensive care or is breastfeeding or taking a bottle (not via the feeding tube) the study will stop and your baby will receive the usual care as they normally would be during feeding.

**7) Who is involved in this study?**

The National Institute for Health Research (NIHR) Health Technology Assessment (HTA) programme is funding the study. The study has been reviewed by the NIHR HTS and the (<INSERT NAME>) Research Ethics Committee (<*insert REC NUMBER>*), who have agreed that the study is being conducted in a correct and appropriate manner. Dr Lyvonne Tume (University of the West of England) is the GASTRIC Study Chief Investigator. The GASTRIC research team are qualified to do this study because they have the specialty knowledge, experience and skills that are needed. Members of team have a lot of experience in caring for children with intensive care and are very active in health research. Parents of babies and children who have experienced being in intensive care have also been involved in the development of this study.

**8) What will happen to the results of this study?**

This study will be presented at conferences and written up for publication in an academic journal but your baby will not be identifiable. Will we prepare a lay summary of the results of this study for all parents who have participated in the trial and we will also place the results on the study website/ twitter page and promote the results via BLISS. We hope that this study will tell us whether not measuring GRV is safe and reduces the time taken for the baby to reach full feeds via the feeding tube.

**9) Will my baby’s taking part be kept confidential?**

Your baby’s study information will be kept completely confidential and stored only on a very secure password protected computer in the trials unit. Only the study team involved in this study will be able to look this information

**10) What if there is a problem?**

**Complaints:** University Hospitals Bristol NHS Trust holds standard NHS Hospital Indemnity and insurance cover with NHS Litigation Authority for NHS Trusts in England, which apply to this study.  If you experience serious and enduring harm or injury as a result of taking part in this study, you may be eligible to claim compensation without having to prove that University Hospitals Bristol NHS Trust is at fault.  This does not affect your legal rights to seek compensation. If you are harmed due to someone’s negligence, then you may have grounds for a legal action.  Regardless of this, if you wish to complain, or have any concerns about any aspect of the way you have been treated during the course of this study then you should immediately inform the Investigator

The normal National Health Service complaints mechanisms are also available to you.

For NHS service advice or support please contact: Patient Advice and Liaison Services (PALS) services. Go to <http://www.nhs.uk/> to find your local PALS contact details. For support on any other issue please contact: BLISS [www.bliss.org.uk](http://www.bliss.org.uk)

**S3 Staff focus group topic guide**

*Italic Black text= Actions/ prompts to facilitator*

Black text =questions

Yellow highlight: Voting handset/ questionnaire

*Give out consent forms/ information sheets*

*With the second slide up:*

*Tell the room: what the GASTRIC study is and that the main aim of the focus group is to find out how you think we should run the GASTRIC study, how it should be designed and how it should run. We will use these findings, alongside the rest of the data collected during the study from the survey and parents, to establish the feasibility of conducting a larger trial. We are doing other focus groups at others sites.*

*We would really appreciate it if you can be as honest as possible – the questions you answer by the voting handsets are anonymous. We will ask you to introduce yourselves to help with facilitation. With your consent, we will audio record this session. An external company called Voicescript analyses the transcripts and we will remove any names, or identifiable information, before analysis.*

Does anyone have any questions before we start?

**Check consent for audio recording – press record**

Practice TP question: if you could have a super power what would it be? Flying/ invisibility/super strength/ X-ray vision

**Section 1: Role and involvement in research**

TP 1: Please tell us what your role is at this hospital: Junior doctor/ Senior doctor/ Junior nurse/ Senior nurse/ Dietitian/ Primarily research nurse/ Other

Please go round the room and introduce yourselves & your role - explore RN full time/ part time/ split with clinical.

TP 2: Are you involved in clinical care of children (e.g. delivering medical interventions?) (Yes/No)

TP 3: Do you have experience of conducting paediatric clinical trials? (0-2 years/2-4/4-6/6-8/8-10/over 10)

**Section 2: Current practice**

Please explain your current approach to measuring GVR

How often do you measure GRV?

Why do you believe we measure GRV?

Do you/parents put the stomach contents back? Or do you or dispose it?) (Explore reasons)

Have you heard of a family integrated care model?

Could you tell me what that involves?

Do you ask parents to measure the GRV? Explore

***Tell them about the proposed GASTRIC study using PIS***

What do you think are the potential ***benefits* of not** measuring GRV?

What do you think are the potential ***risks* of not** measuring GRV?

How would you feel about being part of a trial like this? Explore views

What do you think would be potential barriers to doing a trial like GASTRIC in UK NICUs/PICUs (*as appropriate*)? (Explore and clarify to inform next question)

Is there anything you think could be done by the trial team to have address (X) in preparation for GASTRIC?

**Section 3: Recruitment and consent in the proposed GASTRIC Study**

*This trial could be run using individual randomisation (standard RCT) or using a cluster randomisation method where a group, such as a whole PICU or NICU is randomised to one arm. Both have advantages and disadvantages.*

**Individual RCT:**

If we individual randomised patients to either GRV or NO GRV:

- How do you think you would feel if you were involved in this trial? Explore any concerns about an individual randomised design. Explore any potential benefits of an individual randomised design.
- How do you think parents would react to being asked to participate in a trial like this?
- Would you have any concerns about discussing this trial with parents?

**Cluster RCT:**

*Explain: If the trial was a cluster RCT there would be information available for parents on the unit making it clear that this unit was participating in a trial and their child would receive the intervention, and we would not seek individual parent consent. However due to new GDPR rules parents could out of their child’s data being collection (from that point forward) – but their child would receive the intervention (as the whole unit had been trained in the new method/approach).*

- How do you think you would feel if you were involved in this trial? Explore any concerns about a cluster design. Explore any potential benefits of a cluster design.
- How do you think parents would react to being asked to participate in a trial like this?
- Would you have any concerns about discussing this trial with parents?

TP 4: What randomisation approach do you think we should use in the proposed GASTRIC trial? Individual /Cluster

**Section 4: Potential barriers and compliance**

TP 5: Do you think there will be any barriers to staff **not measuring** GRV? Explore responses Prompt Do you think nurses be willing to actually do this (not measure GRV)? Why or why not.

TP 6: Do you think there will be any barriers to staff **measuring** GRV? Explore responses Prompt Do you think nurses be willing to actually do this (measure GRV)? Why or why not

**Section 5: Inclusion and exclusion criteria and training**

TP 7: Are there patients you think we should definitely NOT include in this proposed trial?

Who?

Why or why not

Do you think this trial should only involve children who are mechanically ventilated?

What education and training do you think staff (maybe nurses in particular) would need to be comfortable in participating in a trial like this?

Do you think nurses would require extra information or training about other clinical parameters to use if they could not use GRV?

If yes, what information would nurses need? How should this be delivered?

Would a flow chart/guideline or algorithm for feeding with no routine GRV be helpful for the nurses?

Would they value a training package (either e learning or other)?

What would help staff to follow a No GRV guideline?

**Section 6: Acceptability**

TP8: Do you think a trial of GRV **measurement** is practically possible to conduct? Yes / no

TP9: Do you think a trial involving **not** measuring GRV is practically possible to conduct? Yes / no

Explore barriers and facilitators to make sure key issues are captured

TP10: Overall how acceptable do you think it would be to conduct the proposed GASTRIC trial?

Very acceptable / acceptable / not acceptable / very unacceptable / Not applicable

*Explore answers*

**Section 7: Anything else**

- **Before we finish, is there anything you think is important for us to know if we conducted GASTRIC study which we have not already covered?**
- **Explain next steps (staff focus groups, interviews with parents, survey of practice complete, Delphi survey- all will feed into final report and recommendations on whether/how this trial should be conducted)**

**S4 Table 1: Approach to qualitative data analysis**

| **Phase** | **Description** |
| --- | --- |
| 1.Familiarising with data | ED (parent data) and LR (practitioner data) read and re-read transcripts noting down initial ideas on key themes |
| 2.Generating initial codes | Initially, two data-coding frameworks (one for parent and one for practitioner data) were developed using *a priori* codes identified from the project proposal and the interview topic guilde. During the familiarisation stage LR and ED identified additional data-driven codes and concepts not previously captured in the initial coding frame |
| 3.Developing the coding framework | 10% of transcripts were double coded; LR, ED and KW all reviewed and discussed both initial coding frames (practitioner and parent). Making notes on any new themes identified and how the framework could be refined. |
| 4.Defining and naming themes | Following review and reconciliation by ED, LR and KW, revised coding frames were subsequently developed and ordered into themes (nodes) within the *NVivo* Database |
| 5.Completing coding of transcripts | LR and ED completed coding of all transcripts in preparation for further analysis |
| 6. Data synthesis | Synthesis of qualitative and quantitative data using the constant comparative method and drawing on the theoretical Framework of Acceptability. |
| 7.Producing the report | ED, LR and KW developed the manuscript using themes that related back to the study aims to ensure key findings and recommendations were relevant to the GASRTIC Study. Final discussion and development of selected themes occurred during the write-up phase. |
